# GPS Digital Nudge to Limit Road Crashes in Non-Expert Drivers

**DOI:** 10.3390/bs12060165

**Published:** 2022-05-27

**Authors:** Raffaella Nori, Micaela Maria Zucchelli, Marco Giancola, Massimiliano Palmiero, Paola Verde, Anna Maria Giannini, Laura Piccardi

**Affiliations:** 1 Department of Psychology, University of Bologna, 40127 Bologna, Italy; micaela.zucchelli3@unibo.it; 2 Department of Biotechnological and Applied Clinical Sciences, L’Aquila University, 67100 L’Aquila, Italy; marco.giancola@graduate.univaq.it (M.G.); massimiliano.palmiero@univaq.it (M.P.); 3 Italian Air Force, Experimental Flight Center, Air Test Division, Aerospace Medicine Department, Pratica di Mare, 00071 Pomezia, Italy; 4 Department of Psychology, Sapienza University of Rome, 00185 Rome, Italy; annamaria.giannini@uniroma1.it (A.M.G.); laura.piccardi@uniroma1.it (L.P.); 5 Cognitive and Motor Rehabilitation and Neuroimaging Unit, IRCCS Fondazione Santa Lucia, 00179 Rome, Italy

**Keywords:** non-expert drivers, driving behaviour, spatial ability, digital nudge, GPS, aggressive violation

## Abstract

Many automotive industries are developing technologies to assist human drivers in suggesting wiser choices to improve drivers’ behaviour. The technology that makes use of this modality is defined as a “digital nudge”. An example of a digital nudge is the GPS that is installed on smartphones. Some studies have demonstrated that the use of GPS negatively affects environmental learning because of the transformation of some spatial skills. The main purpose of this study was to investigate the use of the GPS nudge and its relationship with spatial ability, together with its function in supporting the driving behaviour of non-expert drivers, in order to reduce the number of road crashes. A total of 88 non-expert drivers (M age = 21 years) filled in questionnaires and carried out tasks to measure spatial abilities, sense of direction, driver behaviour, and six different real-life driving scenarios. The results reveal that the higher the spatial skills are, the greater the GPS use is, and that drivers who use GPS improve their sense of direction. Moreover, people with high visuospatial abilities use GPS more extensively. Finally, young drivers do not consider the GPS aid to be useful when they have no time pressure. The results are discussed by taking into account the familiarity-and-spatial-ability model.

## 1. Introduction

The European Transport Security Council [1] declared that there were around 3900 fewer road deaths in the European Union in 2020 compared to 2019 (17% less than the previous years), which was probably due to COVID-19 restrictions on travel. However, the data presented by the European Commission [2] show that, in 2021, over the first six months, the number of road accidents increased (2%), compared to the same period in 2020, even if it substantially decreased considering the previous three-year period of 2017–2019 (19%). Mainly, deaths occur in collisions that involve cars and trucks, but the European Commission [2] has pointed out the need to increase the protection of vulnerable road users, such as pedestrians and cyclists. Three main factors contribute to road accidents: vehicle, human, and road conditions [3,4]. For example, since the 1980s, the causes of vehicle rollovers have focused on injuries or the death of the occupants. Children seem particularly vulnerable to frontal impacts, while travelers over 65 years, compared to those a few years younger, are more at risk of spinal injuries when the impact is frontal, side, or vehicle rollover [5]. For this reason, research has aimed at solving the rollover problems by inserting robust steering control for the vehicle in order to decrease the probability of these types of accidents occurring [6]. 

However, statistics published by the European Commission show that over 90% of traffic accidents were caused by human errors in 2016; high-speed driving and driving under the influence of alcohol or drugs were the three causes that accounted for about 80% of the traffic accidents and deaths in Europe [7]. In addition, the non-use of seat belts contributes to increasing the number of injuries and victims [8].

Generally speaking, driving is an activity that requires visual, motor, and cognitive skills [9,10]. Researchers report that social factors, such as age and gender, and cognitive abilities, such as the visuospatial ability, affect driving behaviour (i.e., [11,12,13,14]). With regard to social factors, young drivers and passengers wear their safety belts less often than old drivers, make more decision-making errors (e.g., inappropriate speed for conditions), and have inadequate surveillance and hazard perception. Moreover, they are often distracted by using the cell phone and by the presence of peers (e.g., [15,16,17,18]). In terms of cognitive abilities, people with high spatial abilities can make correct spatial decisions and travel without incurring violations and fines [14]. This finding is in line with the importance of spatial cognition in daily life and across the lifespan [19,20,21]. Indeed, it is involved in the mental representation of the environment, and in processing objects’ relationships and one’s movements through the world [21]. It is crucial in travel planning, changing destination, and in solving spatial problems. Undoubtedly, the driver’s ability is strongly associated with his/her ability to make simultaneous decisions, maintain attention and control, and quickly process and maintain spatial information online. The driver also needs to mentally transform objects and to continuously update his or her position in the environment [22,23].

There is a shared worldwide effort to reduce road accidents, as their impact on the economy is high. For example, the estimated economic cost of all traffic crashes in the United States in 2010 (the most recent year for which cost data is available) was USD 242 billion. Within the economic costs are included the following: lost productivity, workplace losses, legal and court expenses, medical costs, emergency medical services, insurance administration costs, congestion costs, and property-damage costs [24]. This particularly applies to non-expert drivers. The differences between the two groups are difficult to define. However, for the car civil-liability policy, people under 26 years of age are considered non-expert drivers, according to the insurance contract that covers damage that is involuntarily caused to people or things by the circulation of vehicles. According to some studies, police driving instructors demonstrated safer driver behaviour compared to non-police-trained controls in driving simulator studies [25]. There is, indeed, something qualitatively/quantitatively different in terms of the driving skills of expert drivers: they, in fact, exhibit observational skills that are indicative of driving schemas that are more detailed and nuanced compared to those of non-expert drivers [26,27]. Specifically, expert drivers allocate less attention to basic operational tasks (i.e., maintaining the car’s position within the carriageway) when compared to the non-expert, and this behaviour suggests that actions are automatized in conjunction with the construction and enhancement of a well-defined driving schema [28]. For these reasons, non-expert drivers are nearly four times more likely to be involved in an accident per mile than drivers aged 20 years or over [29]. Yet, the probability that a fatal accident occurs is also higher than in any age group, except for the over-80s. This is due—explains the report—to a set of risk factors, including speeding, the failure to use seat belts, and inexperience.

After this brief introduction, we propose the state of the art in the use of GPS.

We describe the participants and materials. Then, we report the data analysis and results. Finally, the discussion, conclusion, and future studies to face the problem are presented.

### 1.1. State of the Art

Governments and automotive industries demand studies or developments in assistance systems to help drivers in order to prevent accidents and, consequently, to avoid a waste of time and money. Currently, many automotive industries are developing technologies to assist human drivers in suggesting wiser choices without limiting any of the options to improve drivers’ behaviour [30]. The technology that uses this modality to suggest less risky and wiser behaviour is defined as a “digital nudge” [30,31]. In order to improve road safety, over the years, a lot of digital-nudge interventions have been developed. As a rule, these interventions can be divided into increasing safety levels by assisting the driver (Advanced Driver Assistance Systems [32]), or in improving road safety by applying perceptual elements, such as optical speed bars, optical circles, and so on (e.g., [33]). This approach seems to be very promising: the use of digital nudges is useful in reducing road crashes when interventions do not produce any negative side effects (e.g., [34]). The GPS that is installed on smartphones is an example of a digital nudge: the individual decides where she/he wants to go, and the application offers possible routes, but the individual is free to refuse suggestions if she/he decides to make a detour [31].

Navigation technologies that use the GPS have become very popular among car drivers because they assist drivers by indicating the direction to the destination from their current position and perspective, which consequently reduces the effort in acquiring, processing, and learning spatial knowledge, and they indicate the time that is necessary to reach the goal, the speed limits, the speediest road, the toll roads, and how to avoid one-way streets or those that are closed for work in progress, (e.g., [35]). Because of the fact that the GPS indicates the route to travel from the current position to the goal, and it updates the travel perspective, it *de facto* supports people in acquiring, processing, and learning spatial knowledge and helps them to build a spatial environmental representation (e.g., [36,37]). It is important to point out that driving without GPS technology is an active spatial cognitive task (the driver has to process spatial information, memorize the route, and decide where to turn), while driving with GPS technology is a passive driving behaviour because the tasks are executed under audio-visual guidance instructions [35,38]. Consequently, the use of GPS permits a better wayfinding performance (that is, the ability to reach the goal) [36]. However, some data do not support this evidence and show the negative effects caused by the prolonged use of GPS. For example, Ruginski and co-workers [39] demonstrated that GPS use negatively affects environmental learning through some spatial-transformation skills. Specifically, they observed that mental rotation mediated the negative effects of GPS use on perspective-taking, which, in turn, conveyed this effect on the measures of environmental learning. In addition, Dahmani and Bohot [40] show, by a longitudinal study, that GPS use was associated with a steeper decline in spatial memory. Interestingly, He and Hegarty [41] also show that people with low spatial anxiety rely less on GPS during navigation. This led to the supposition that, despite the extended use of GPS negatively affecting spatial abilities, it is also likely that a low spatial ability can predict the extensive use of GPS. Thus, it becomes difficult to understand whether one is the effect of the other. What is the question? Do I use the GPS more because I have low visuospatial skills—which, in turn, will not allow me to improve because I passively use a tool and do not acquire an active knowledge of the environment—or does the use of GPS reduce my skills even more? The most relevant results of these studies are systematised in Table 1.

To the best of our knowledge, the use of GPS in decreasing dangerous driving behaviour, and specifically for non-expert drivers, has never been analysed. Therefore, the main objective of this manuscript is to explore whether the different spatial abilities may predict the use of the GPS nudge to limit the risk assumption in the driving behaviour of non-expert drivers. As shown by Nori and co-workers [14], drivers differ in their behaviour also because of navigational strategies, and, in particular, individuals with better navigational skills and, consequently, good visuospatial skills, are better at making spatial decisions and are more confident about their spatial competence, and this makes them less aggressive towards other drivers, and less prone to incur violations and fines. However, in the study by Nori and co-workers [14], the relationship between the visuospatial skills and GPS use in non-expert drivers was not investigated.

### 1.2. Aims and Hypothesis

The present work aims to fill this gap through a threefold objective.

The first aim is to analyse the differences in the visuospatial abilities of non-expert drivers in the use of GPS. Specifically, we hypothesise:

**H1.**  *Non-expert driverswith low visuospatial abilities are more prone to use a GPSnudge, which is aimed at assisting people while driving by helping them to not become lost in the environment and to avoid violations and fines, and vice versa [42]*.

A further aim is to investigate the effect of GPS use on the sense of direction (SOD) and the town knowledge (TK). Specifically, we hypothesise:

**H2.**  *GPS users develop a better SOD and a different TK than GPS nonusers.*

Moreover, we analysed the driving behaviour of GPS users, and we hypothesise:

**H3.**  *GPS users, by using a passive strategy to drive, perform less ordinary violations, lapses, and errors.*

Finally, we analysed real-life driving scenarios to better understand the context effect on the use of GPS.

**H4.**  *Regardless of the visuospatial abilities, time-pressure scenarios induce a higher use of GPS than no-time-pressure scenarios.*

## 2. Materials and Methods

### 2.1. Participants

Our sample consisted of 88 participants [GPower 3.1; Faul et al. [43]: f2 = 0.15, α = 0.05, power = 0.90], who were recruited at an Italian university campus and from cultural associations through notices on social networks and on bulletin boards. The inclusion criteria were: regularly driving a car with a B license guide (licensing information: http://www.mit.gov.it/come-fare-per/patenti-mezzi-e-abilitazioni/patenti-mezzi-stradali; accessed on 25 January 2016); being non-expert drivers (drivers between 18 and 26 years of age are not experienced, according to insurance companies: mean of driving months = 31.00; SD = 24.61). We used a sample with a mean age of 21 years (SD = 2.12; 33 males; the education level is: M = 14.38, SD = 2.11). Exclusion criteria were: history of neurological or psychiatric disorders or abuse of alcohol/drugs. No participants were excluded. All participants signed the informed-consent form to participate in the study. The study was designed in accordance with the latest version of the Declaration of Helsinki and was approved by the local Ethics Committee (Prot. n. 118410 of the 30 May 2019, University of Bologna, Bologna, Italy).

### 2.2. Material

All the participants filled in the following questionnaires: 

***Familiarity and Spatial Cognitive Style Scale—Short Version*** (FSCS) [44,45,46]. The short version of the FSCS [45] consists of 9 questions: 5 items (1–2–4–5–8) provide a general evaluation of participants’ sense of direction (SOD) (i.e., How is your SOD?), and 4 questions (11–12–13–14) evaluate participants’ town knowledge (TK) (i.e., How well do you know Bologna?). Participants respond by rating items on a 5-point Likert scale (from 1 = very poor to 5 = excellent).

***Spatial Cognitive Style Test—Short Form*** (SCST) [47,48]. In order to assess the individual’s spatial ability, the short version of the SCST [45] was used. It is composed of 6 tasks:

*Photo task*. The participants were asked to memorize a photo of a building for 3 s. Afterwards, they had to recognize the target building among four fillers;

*Figure task*. Participants had to study seven geometric figures for 75 s, and, immediately after, they were asked to recognize them among 50 shapes (7 targets and 43 fillers);

*Sequence task.* Participants were asked to study a photo representing an environmental scene from a first-person perspective for 15 s. Then, the environmental scene was divided into separate panels (3, 4, or 5). The participant’s task was to reconstruct the previous picture by assembling all the parts in the correct sequence from left to right;

*Map Description task*. Participants were asked to describe a route drawn on a map; starting from a black starting point, they had to describe the path to reach a red goal point by reporting the correct sequence of seven right–left turns. Participants were allowed to rotate the sheet of paper on which the map was printed in order to ensure that only right and left discrimination and sequential abilities were involved;

*3D Rotation task.* Participants were required to mentally rotate a figure of an old TV in the direction indicated by one or two arrows following four possible rotations, and then were asked to choose the resulting position among five alternatives;

*Sum and Straighten task*. A series of three segments were shown to the participant, who were instructed to mentally sum and straighten them, and to select the resulting segment among four alternatives.

The order of the six tasks was randomized. As in Boccia et al. [49], we computed an aggregate score that resulted from the sum of the tasks’ scores (from 0 to 42). The aggregate score (AS) may be considered as an index of the participants’ spatial ability, and it allows for the maintenance of the individuals’ spatial abilities on a continuum.

***Manchester Driver Behaviour Questionnaire*** (DBQ) [50].

In order to assess the driving behaviour of participants, the Manchester Driver Behaviour Questionnaire (DBQ) [50] was used. It is a self-report questionnaire, which measures driving behaviour in terms of (i) *lapses* (e.g., How often do you hit something you did not see when you turn around?); (ii) *errors* (e.g., How often do you manoeuvre without checking mirror?); (iii) *Highway Code violations*, that is, *ordinary violations* (e.g., How often do you race away from traffic lights with the intention of beating the driver next to you?); and (iv) *aggressive violations* (e.g., How often do you sound your horn to indicate your annoyance to another driver?). Participants were required to indicate, on a six-point scale, ranging from ‘‘never to” (1) to ‘‘nearly all the time” (6), how often they committed that specific behaviour while driving. The internal-consistency reliability (Cronbach’s alpha) for our sample is 0.72 for the four scales of the questionnaire.

***Scenarios***

Six different scenarios were created in order to evaluate whether participants would follow navigator instructions or not in 3 contextual situations: (i) *intense road traffic* vs. *flowing traffic*; (ii) *familiarity or not with the route to go through*; and (iii) *the* *presence or not of a time pressure* due to being on time or late for an appointment. For each scenario, the participants were required to decide whether or not to follow the GPS-nudge suggestion to reach the goal. The six scenarios were tested in a pilot experiment, and they were developed on the basis of previous studies about nudge plans (e.g., [51,52]). We asked 19 participants (mean age = 25.15 ± 3.40 years; mean education = 15.15 ± 2.40 years) to evaluate the *degree of involvement,* on a scale between 1 (not at all) and 10 (high involvement), of the scenarios, in order to exclude differences in terms of emotional involvement with the situation. Results show that all six scenarios were comparable in terms of emotional involvement (F_5.113_ = 0.62, *p* = 0.67, ƞ_2_ = 0.02), and so they were used in the following study. The answer was binary: the decision to accept the GPS nudge was coded as “1”, while the decision not to accept was coded as “0”.

### 2.3. Procedure

Before starting the experimental session, the participants signed a written informed-consent form and filled out a questionnaire on basic demographics (gender, age, and education) and driving-habit information, including an estimation of how many kilometres they drive weekly, in order to be sure that participants had the same driving experience. Then, they filled in all the questionnaires and answers to the scenarios, which were submitted in a randomized order, with the exclusion of questions about the use of the GPS nudge, which were always collected at the end of the experiment to avoid any influence on answers provided to the scenarios. Each participant was instructed to accurately reflect while responding about questions on his/her SOD and TK (Familiarity and Spatial Cognitive Style Scale—Short Version (FSCS) [42]). The same occurred for the Manchester Driver Behaviour Questionnaire (DBQ) [50]. Answers were collected on a personal computer and the experimenter remained available for questions or doubts. With regard to the SCST [47], the task was administered by the experimenter, who collected the answers provided by the participants. Then, the scenarios were administered in random order, and the participants were instructed to imagine being in the context described, and to try to provide answers, as much as possible, that were close to their actual decisions in real life. The whole experiment lasted approximately 1 h.

## 3. Results

In order to investigate, in non-expert drivers, the relationship between the visuospatial abilities in the use of the GPS in everyday life, we performed a regression analysis that considered the aggregate scores (AS) of the SCST’s tasks as a measure of the visuospatial abilities, and the frequency of the use of the GPS (H1). The results show that those who had higher visuospatial abilities chose to use the GPS more. The statistics are detailed in Table 2.

In addition, to investigate the effect of the GPS use on the SOD and TK, we performed two regression analyses that considered the use of the GPS nudge as a predictor of *SOD* (mean score of FSCSQ) and *TK* (mean score of FSCSQ), respectively (H2). The results show that the GPS nudge positively predicted both SOD and TK. The statistics are detailed in Table 2.

Then, to analyze the influence of the use of the GPS nudge on driving behaviours, we performed a series of linear regressions that considered the use of the GPS nudge as a predictor, and the ordinary and aggressive violations, errors, and lapses that were measured with the Manchester Driver Behaviour Questionnaire (DBQ) [50] as the dependent variables, respectively (H3). The only significant result was with regard to the aggressive violations, which indicates a positive association between the use of the GPS nudge and aggressive violations. The statistics are detailed in Table 2.

**Figure 1:**
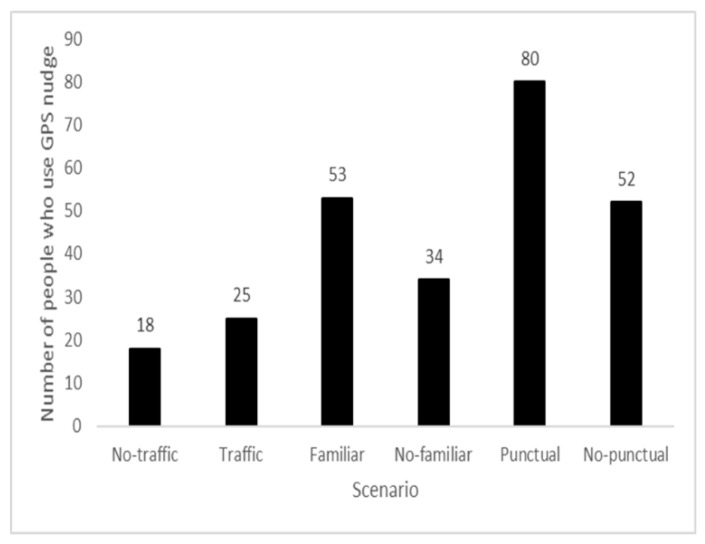
The numbers of people who used GPS nudge in the different scenarios.

Finally, to evaluate whether there are external conditions (kind of scenario) that elicited the use of GPS (H4), we performed a Cochran Q test. We classified 1 when a person uses the GPS nudge, and 0 when a person does not use GPS. The analysis revealed a significant difference among the 6 scenarios (Q_5_ = 112.05, *p* < 0.001). The pairwise comparisons showed that the no-traffic scenario differed by the familiar-path (*p* < 0.001), non-familiar-path (*p* < 0.05), punctual (*p* < 0.001), and non-punctual (*p* < 0.001) scenarios; the non-traffic scenario differed by the non-familiar-path (*p* < 0.05), punctual (*p* < 0.001), and non-punctual (*p* < 0.001) scenarios; the non-familiar-path scenario differed by the familiar-path (*p* < 0.01), punctual (*p* < 0.001), and non-punctual (*p* < 0.001) scenarios; and the punctual scenario differed by the non-punctual (*p* < 0.001) scenario. See the descriptive in Figure 1.

## 4. Discussion

The main purpose of the present study was to investigate the use of the GPS nudge to support the driving behaviour in non-expert drivers in reducing the number of road crashes. Our results partially support our hypothesis. As far as the relationship between spatial abilities and the use of the GPS nudge is concerned, our results reveal that the higher the spatial skills are, the greater is the GPS use. It would seem that those who have higher visuospatial abilities are more prone to move autonomously in the environment and to explore unknown roads, at least by car, and this leads them to make greater use of a tool that makes it easier for them to navigate the environment. Moreover, the use of the GPS nudge predicts SOD and TK. Drivers who use GPS improve their SOD. Of course, we cannot conclude that this is always true with respect to the long-term use of the GPS nudge, as reported by Ruginski and co-workers [39]. Undoubtedly, we found that, at the beginning, the GPS nudge produces a positive effect on SOD. Moreover, we also found that drivers who more frequently use the GPS nudge acquire a better representation of the town, which, in turn, helps them to move successfully and on time through the town. As noted in the Environmental Knowledge Model [53], familiarity with the environment is the most important internal factor that is capable of attenuating or eliminating the spatial differences that likely affect driving behaviour. Several studies demonstrate that poor navigators with high environmental familiarity may also perform very complex navigational tasks [54,55,56]. Consequently, familiarity with the environment reduces the importance of the layout complexity [54]. The familiarity, indeed, impacts not only the environmental knowledge, as town knowledge, but also on the SOD (e.g., [53,57]).

These findings are confirmed by the driving behaviour: people with high visuospatial abilities move more often in unfamiliar places and tend to use the GPS more frequently in order to reach their destination with certainty, without incurring traffic violations and fines. Probably, drivers with poor visuospatial skills tend to explore new environments less and often drive on the same roads. This type of behaviour does not lead to an increase in the representation of the town, which remains restricted to the places they usually explore. As pointed out by Denis et al. [58], when people with poor spatial skills have to run across a new road, they do not know how to reach the destination and they proceed by trial and error until they memorize a path, and then they always use it. A typical example is the route from home to work, which becomes over-learned to the point of knowing exactly where the speed controls are located and so on; in this case, individuals do not need to use a GPS nudge, which is instead used when there is a need for making a detour. Generally speaking, the use of the GPS nudge facilitates driving by informing drivers, in real time, about the speed limits, the presence of speed control, and obstacles on the road; nevertheless, people with poor visuospatial abilities and a more habitual driving style tend to use the GPS nudge to a lesser extent, and to not move in unknown places in this way, and they also do not incur traffic violations, but for different reasons.

As far as the result about aggressive traffic violations (i.e., sounding your horn to indicate your annoyance to another driver, becoming angered by another driver and giving chase with the intention of giving him/her a piece of your mind, and so on…), a possible explanation may be similar to that which is provided by Perepjolkina and Reņģe [59], who indicate that young drivers usually commit more aggressive violations. Indeed, our sample included only young drivers. Other studies have concluded that the driver’s age is a crucial variable, and that young drivers are more aggressive and risk-oriented than older drivers (e.g., [60,61]). Although our data would seem to indicate that GPS-nudge use encourages aggressive driving behaviour, it does not predict mistakes, lapses, and ordinary traffic violations. This would suggest that GPS use induces good driving behaviour, and that the presence of aggressive behaviour is linked to the young age of our sample.

In the present study, we considered different scenarios of real-life situations (e.g., a no-traffic scenario: no traffic and arriving a few minutes later without complications) and the use of the GPS nudge. We found that young drivers did not consider the GPS to be useful in some situations, such as when there was not time pressure. In this case, even when navigating in an unfamiliar environment, if they did not have a timetable to follow, they decided not to use it. With respect to the scenarios, it emerges that there is a more frequent use of the GPS nudge in the familiar environment, which might appear unusual, but the scenario highlights, specifically, that the use of the GPS nudge helps the driver to arrive at home on time by avoiding traffic. This result is also in line with a study by Tinella et al. [62], who observed that drivers with high visuospatial skills were less stressed by traffic. We can add that they are probably more resilient to traffic because they activate problem-solving strategies, such as activating the GPS and looking for an alternative route without traffic. Moreover, Tinella et al. [62] point out that such resilience was negatively affected by age and positively affected by overall cognitive functioning, gender, and spatial skills. In a recent study, Fillekes and co-workers [63] propose a conceptual framework that shows the analytic aspects that inform how and when a GPS nudge could be used. Their results have uncovered some of the dimensions that are required to obtain a comprehensive view of an adult/older person in using the GPS nudge during daily mobility, which include: space (i.e., including distance travelled, complex mobility environment), time (i.e., time spent out of the home and the activity distribution during the day), and movement-scope categories (i.e., trips between the locations). Our results better specify the space and time categories: in the first case, when the space, which is intended as an environment in which people travel, does not have traffic, people tend to not use the GPS nudge; this is different from the time category, in which, when people need to be punctual for an appointment and they want the certainty of being on the spot, they activate the GPS nudge.

### Conclusions and Future Works

In conclusion, our results show that, in the non-expert-driver population, the GPS nudge is mostly used by those with high visuospatial skills, since they explore more unfamiliar places and roads and, therefore, the GPS nudge is used to reduce the number of ordinary traffic violations and fines. Drivers with low visuospatial skills have the same number of traffic violations as high-skill drivers, even if they do not use the GPS nudge. This is probably due to the fact that they drive in familiar places and they know the traffic conditions very well. However, regardless of spatial skills, the GPS nudge is mostly activated when people need to arrive punctually. Future studies should test a different population, including drivers with very different levels of expertise, to better understand when GPS-nudge use is suitable also for people with poor spatial skills. Furthermore, our data do not allow us to understand whether or not the use of GPS can be advantageous for the elderly population, considering that visuospatial and navigational skills are the first to be affected by ageing [64,65,66,67,68,69]. Furthermore, old people have resistance to the use of technology, but, considering that GPS is a passive tool, this population could benefit from it. In fact, as demonstrated by Nori and co-workers (2021), the difference between the old and the young in the use of technology concerns the active aspects; therefore, in the use of GPS, there should be no differences. Nevertheless, this issue should be better investigated in future works.

It could be very interesting also to investigate the use of the GPS nudge in clinical populations that suffer from navigational disorders to better understand if the GPS nudge could help them to incur traffic violations to a lesser extent. A GPS-nudge system with a simplified interface and with clearer and more immediate visual and auditory indications could be useful to this specific population and, more in general, to people with poor visuospatial skills.

## Figures and Tables

**Table 1:** Main studies concerning the relationship between GPS use and visuospatial abilities.

Authors	Aims	Findings
Ishikawa et al. [36]	Comparison among GPS, paper maps, and direct experience of a route on wayfinding and spatial knowledge	GPS users have worse performances than direct-experience participants
Ruginski et al. [39]	GPS use is associated with worse mental spatial-transformation ability in acquiring the virtual environment	GPS users have worse performances in perspective-taking and in mental rotation of the acquired virtual environment
Dahmani and Bohbot [40]	Longitudinal study to analyse the effect of extensive GPS use on visuospatial ability	GPS use leads to a decline in spatial memory
He and Hegarty [41]	Explore the tendency to rely on GPS during navigation on the spatial ability of people with spatial anxiety	People with low spatial anxiety tend to explore the environment extensively and tend to use GPS less

**Table 2:** Summary of regression-analysis values based on the three hypotheses formulated (SOD = sense of direction; TK = town knowledge; DB = driving behaviour).

	Predictor	β	*p*	R	R^2^	F_(df)_
H1	Visuospatial abilities	0.27	*p* = 0.01	0.27	0.07	7.02_(1.87)_
H2	SOD	0.20	0.05	0.20	0.04	3.81_(1.87)_
	TK	0.33	*p* = 0.001	0.33	0.11	10.82_(1.87)_
H3	DB: aggressive violations	0.22	*p* = 0.03	0.22	0.04	4.42_(1.87)_
	DB: ordinary violations	0.07	*p* = 0.47	0.07	0.00	0.51_(1.87)_
	DB: errors	−0.06	*p* = 0.57	0.06	0.00	0.31_(1.87)_
	DB: lapses	−0.008	*p* = 0.94	0.00	0.00	0.005_(1.87)_

## Data Availability

The data presented in this study are openly available in a repository OSF at https://osf.io/xbzgy/?view_only=a76093a671b74287b523a3721be2a95b (accessed on 13 May 2022).
